# Phosphorole

**Published:** 1879-11

**Authors:** 


PHOSPHOROLE, their new combination of phosphorus with cod liver oil, appears to be all that has been claimed for it. We trust a thorough trial of its virtues in phthisical cases will be made by some of our correspondents, and that they will furnish us complete reports for publication.

